# Mutation Rates and Evolution of Multiple Coding in RNA-based Protocells

**DOI:** 10.1007/s00239-014-9648-6

**Published:** 2014-10-04

**Authors:** Folkert K. de Boer, Paulien Hogeweg

**Affiliations:** Theoretical Biology and Bioinformatics, Universiteit Utrecht, Utrecht, The Netherlands

**Keywords:** RNA evolution, Genotype-phenotype mapping, Mutation rates, Genome structure, Information threshold, Origin of life

## Abstract

RNA has a myriad of biological roles in contemporary life. We use the RNA paradigm for genotype-phenotype mappings to study the evolution of multiple coding in dependence to mutation rates. We study three different one-to-many genotype-phenotype mappings which have the potential to encode the information for multiple functions on a single sequence. These three different maps are (i) cofolding, where two sequences can bind and “cofold,” (ii) suboptimal folding, where the alternative foldings within a certain range of the native state of sequences are considered, and (iii) adapter-based folding, in which protocells can evolve adapter-mediated alternative foldings. We study how protocells with a set of sequences can code for a set of predefined functional structures, while avoiding all other structures, which are considered to be misfoldings. Note that such misfolded structures are far more prevalent than functional ones. Our results highlight the flexibility of the RNA sequence to secondary structure mapping and the power of evolution to shape the genotype-phenotype mapping. We show that high fitness can be achieved even at high mutation rates. Mutation rates affect genome size, but differently depending on which folding method is used. We observe that cofolding limits the possibility to avoid misfolded structures and that adapters are always beneficial for fitness, but even more beneficial at low mutation rates. In all cases, the evolution procedure selects for molecules that can form additional structures. Our results indicate that inherent properties of RNA molecules and their interactions allow the evolution of complexity even at high mutation rates.

## Background

The RNA-model can be used not only to unravel the role of RNA in the evolution of complexity, but also helps in identifying important general properties of information processing, i.e., genome architecture and its mapping to functions. As such, the genotype-phenotype mapping of RNA is considered to be a paradigm model to study the evolutionary processes (Fontana and Schuster 1998; Fontana 2002).

In the light of bioinformatic processes, RNA was long considered to be only an intermediate molecule, translating genetic information, stored on DNA, into functional proteins. Yet, besides a plethora of new functions of non-coding RNA (not encoding proteins) which have been discovered in the past years (Bompfünewerer et al. 2005), more and more evidence is revealed about a layer of regulation largely consisting of RNA, which actually governs information to function processing. That is, most cellular processes may be modulated by micro-RNAs (van Kouwenhove et al. 2011), and it is generally accepted that phenotypic divergence in animals is based not only on the divergence of genetic information itself, but also on the variation of the regulatory information that controls the expression (Mattick et al. 2010). In other words, the already complex mapping between information and functionality is often also subject to modifications and/or dependent on interactions between molecules.

In addition, in contemporary organisms, the use of information is not as straightforward as originally thought: genetic information is frequently arranged in an interleaved fashion in both DNA and RNA, and two or more transcripts from the same locus might use a common sequence in different ways, to perform distinct biological roles (Tuck and Tollervey 2011). For example, next to coding and non-coding RNAs, bifunctional RNAs also exist;these RNAs carry both RNA-translatable and RNA-intrinsic functions (Ulveling et al. 2011) and RNAs may have multiple functions (Dinger et al. 2011). Moreover, functions can be coded as alternative conformations of a single RNA sequence. Such alternative conformations of RNA are known to be selected for, and thus likely play functional roles in, even the most structured of RNAs (Ritz et al. 2013). Collectively, we refer to such phenomena as multiple coding.

In this paper, we investigate whether the propensity of RNA for multiple coding and as a modifier of information expression could have had a role in early evolution as well. It is well known that genome size is severely constrained at high mutation rates (Eigen 1971). If a simple one-to-one genotype-phenotype map is assumed, functionality is also severely constrained at high mutation rates. The research presented here will explore to what extent the flexibility of the RNA genotype to phenotype mapping can alleviate this constraint. We will refer to genotype-phenotype map flexibility as the range of possibilities determined within a predefined genotype-phenotype map to alter the mapping between genetic information and function. In particular, we are interested in the role of one-to-many mappings of RNA in the evolution of multiple functions in early evolution. In other words, we will explore the evolution of “coding structures” as a function of mutation rates, where coding structure refers to how functionality is coded for on a genome, and the mapping from this code to (a) particular function(s).

To address this question, we consider the evolution of abstract protocells, consisting of RNA sequences, which can attain fitness by the ability to generate a particular set of RNA secondary structures (and avoid all other structures). With these protocells, we can dissect different RNA genotype-phenotype mappings and determine their effects that could influence functional diversity and fitness. We consider three different genotype to phenotype mappings which allow multiple coding, namely adapter-based folding, suboptimal folding, and cofolding. In adapter-based folding, the folding of RNA can be modified by binding to an “adapter” molecule. Recently, we showed that the inclusion of such adapter-mediated alternative foldings (through evolving RNA-adapters) can lead to a complex multiple coding structure and a high degree of functionality also at high mutation rates (de Boer and Hogeweg 2010; de Boer and Hogeweg 2012). Yet, besides this explicit mechanism for one-to-many coding, RNA itself already has the propensity for multiple coding in several ways, i.e., by the ability of RNA to adopt alternative (energetically suboptimal) states and the ability of RNA sequences to cofold with each other. Here, we investigate whether these inherent mechanisms of RNA for multiple coding provide comparable results as adapter-based folding for evolving a coding structure such that high functionality can be attained for a broad range of mutation rates.

Importantly, we explicitly address the possible risks of multiple coding. While on the one hand, multiple coding has been recognized as possibly one of the key features for evolvability through the variability it can provide on a phenotypic level (Ancel and Fontana 2000), the risks have been recognized as well. RNA must avoid the problem of folding into non-functional structures (Herschlag 1995), and it has to do so in a highly crowded cellular interior (Ellis 2001), where molecules are prone to inappropriate interactions with other molecules (Dobson 2003). Longer RNA sequences have a higher propensity of alternative foldings (misfoldings) and may therefore, be disadvantageous despite their higher evolvability (Lorsch 2002). In our model, we therefore, set a dual requirement for high fitness, i.e., maximizing the set of (predefined) functional structures that can be generated, while avoiding all other structures (misfoldings). Note that the set of functional structures is very small relative to the set of misfolded structures.

## The Model

We study a minimal model to investigate the role that multiple coding might have in accumulating functionality despite high mutation rates. The model consists of a population of ‘protocells’ that consist of a variable number of RNA sequences. The secondary structure of the sequences determines functionality and/or toxicity (by misfolding) of the sequences. Selection of protocells takes place to maximize the number and quality of available functional folds and minimize misfoldings. For simplicity (and in line with most previous studies on the impact of mutation rate on information accumulation (but see Ancel and Fontana (2000)), we ignore the kinetics of the folding and of the replication of RNA sequences and protocells. Details of the model are as follows:

### Protocell Genotype

Collection of RNA sequences of variable length (initiated at *L* = 50 ± 10, *N* = 5).

### Protocell Phenotype

**Fig. 1:**
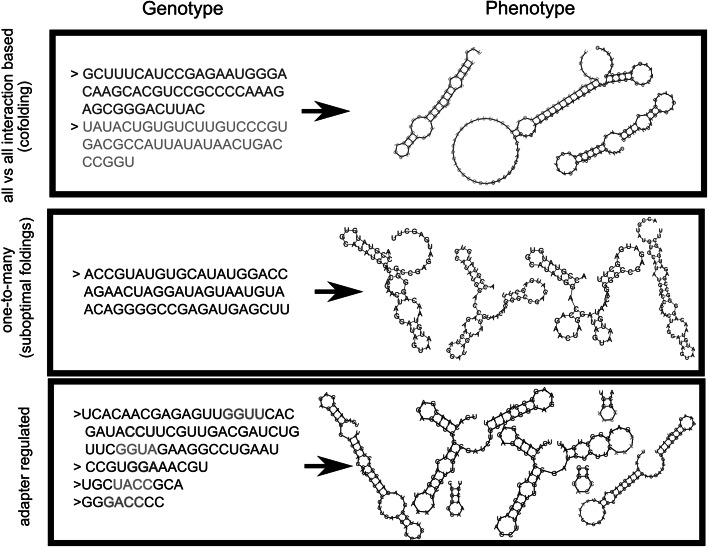
An example for each of the different genotype-phenotype mappings with the phenotype produced by a genotype. Genotype refers to all the information kept in the protocell, i.e., the different RNA sequences. Phenotype refers to all the structures which can be produced with the folding-rules given by the genotype-phenotype map. Cofolding has 2 RNA sequences, which combine in this case into three different structures. The suboptimal folding example has one sequence which has four alternative structures. The adapter-based folding example has three adapters and one ‘normal’ sequence. Corresponding binding sites are colored, which result in three structures next to the native fold

Collection of RNA secondary structures. The secondary structures are characterized by (1) their course-grained Shapiro structure (Shapiro 1988) to determine functionality, (2) the full secondary structure to determine quality of function in terms of distance to the target structures (using the tree-based distance measure defined in the Vienna package), and (3) their free energy. Three different folding protocols for calculating the phenotype are used (see Fig. 1). In all cases, the minimal energy folding (MFE) of each sequence is included.

- *Sub*-*optimal energy folding* (Vienna package (Wuchty et al. 1999; Lorenz et al. 2011)): The folds within .5 kcal/mol of the minimal energy are included. Relative frequency is ignored.
- *Cofolding* (Vienna package (Bernhart et al. 2006)): Each pair of sequences is cofolded and the resulting secondary structure is added if differing from the concatenation of the single sequence folds (i.e., MFE of cofold smaller than the sum of MFE of each sequence). Because the Shapiro structure is order sensitive regarding the cofolded sequences, only the one folding into a functional structure (if any) is taken into account.
- *Adapter-based folding* (Vienna package (Hofacker et al. 1994; Lorenz et al., 2011), (de Boer and Hogeweg 2012)): An ‘adapter’ is predefined as a single hairpin loop. It shields the nucleotides to which it binds maximally (and with binding < −4.0 kcal/mol) from within sequence binding. The resulting secondary structure of the bound/modified RNA is added to the collection. The adapter itself is neither functional, nor toxic.

To assess the potential of each of these folding protocols, they are studied separately. In addition, the combination of the separate regimes is studied. However, because of computational intractability, no full combinatorial combination is considered, i.e., suboptimal foldings of cofoldings and adapter-based foldings are not considered.

### Selection

**Fig. 2:**
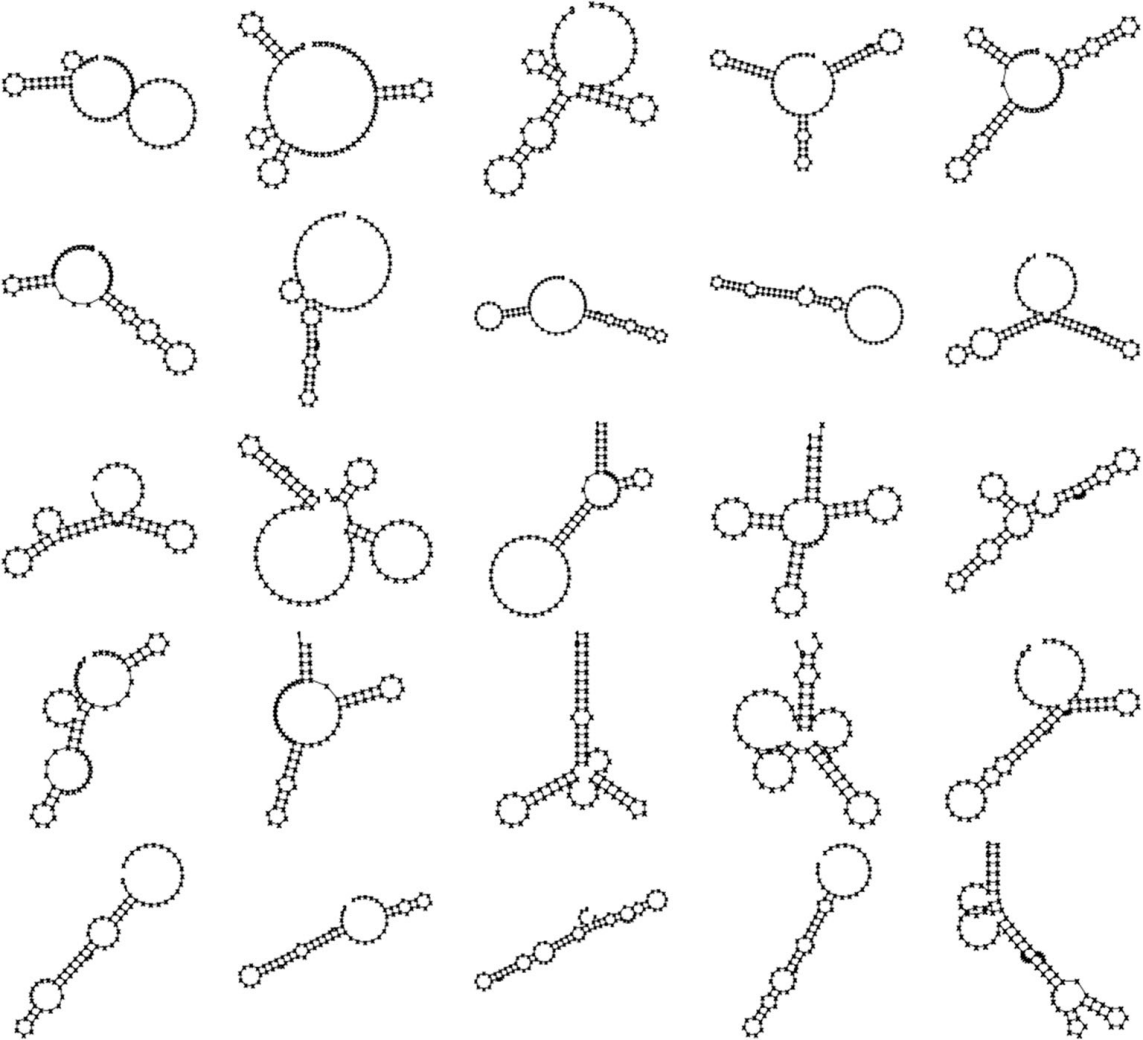
All the used target structures. Exact fitness is based on matching these structures (after removing dangling ends). However, all secondary structures with the same course-grained structure are considered functional. In our earlier work, we compared this set with a random set, leading to similar results (de Boer and Hogeweg 2012). The number of targets is chosen to be slightly larger than the maximum that can be retained at the lowest mutation rate considered. This choice is not structure specific: different structures are chosen in different simulations

Selection is based on two properties of the phenotype, that is on the collection of functional secondary structures and on the collection of misfoldings. Secondary structures are defined to be functional if their course-grained structure matches a Shapiro structure from a predefined set of functional structures. This set (see Fig. 2) was a priori selected, with being ‘different’ as the main criterion and earlier work indicates that this does not influence the results qualitatively (de Boer and Hogeweg 2012). When several RNA structures match the same target structure, only the one with the least energy is taken into consideration. Its contribution to fitness is proportional to the distance of the full secondary structure without its dangling ends to the target structure.

For proteins aberrant foldings are often toxic, and aberrant RNA-foldings can interfere with proteins. We transfer this property here to RNA-only evolution, in order to consider a worst-case scenario for evolving specific functionality. Thus, any folds that do not match the target set as course-grained structures are considered as a misfolding or ‘toxic’. For the ease of implementation and evaluation, we separated these two aspects in different parts of the selection function. The matching to the functional set determines the chance of reproduction (referred to as reproductive fitness or fitness for short), while the mismatch (misfolding) determines the decay.

### Reproduction and Decay of Protocells

In this study, the reproductive units are the protocells, not the individual RNA strings. This is in contrast to many previous models which studied multilevel selection (e.g., the error correction model of Szathmáry and Demeter (1987), or stochastic corrector mechanisms described in Hogeweg and Takeuchi (2003); Takeuchi and Hogeweg (2009)). Here, we are interested instead in multiple coding of a set of RNA sequences that are reproduced together. At reproduction, a new protocell is created with copies of the RNA sequences of the parent protocell subject to point mutations, small insertion/deletion, and duplication or loss of RNA sequences. Protocells decay with a probability *d* + *nt*, where *n* is the number of misfolded structures, and *d* = .4 and *t* = .02 are the fixed decay and the extra decay due to misfoldings, respectively. This fairly low value of *t* is sufficient to result in strong selection against misfoldings.

### Population

We consider a spatially embedded population of a maximum of 50 × 50 protocells. Protocells compete locally for resources (here empty space), on the basis of their reproductive fitness *f*, given by the number of functional RNAs and their relative distance to the targets. Strong selection is used between 8 neighbors and the chance for each neighbor to win the competition is defined as $P_{i} = \left( {\frac{{f_{i} }}{{\sum\limits_{j = 1}^{8} {f_{i} + 1} }}} \right)^{3}$.

### Analysis

To characterize the outcome of the evolutionary process, we focus on the structure of the ‘last common ancestor’ (LCA). The LCA is found by the backtracking of the extant population at *t* = 500000. In practice, this LCA is found within a 1,000 generations. That is, the populations are evolutionarily converging quickly.

## Results

### Fitness is Almost Independent of Mutation Rates

**Fig. 3:**
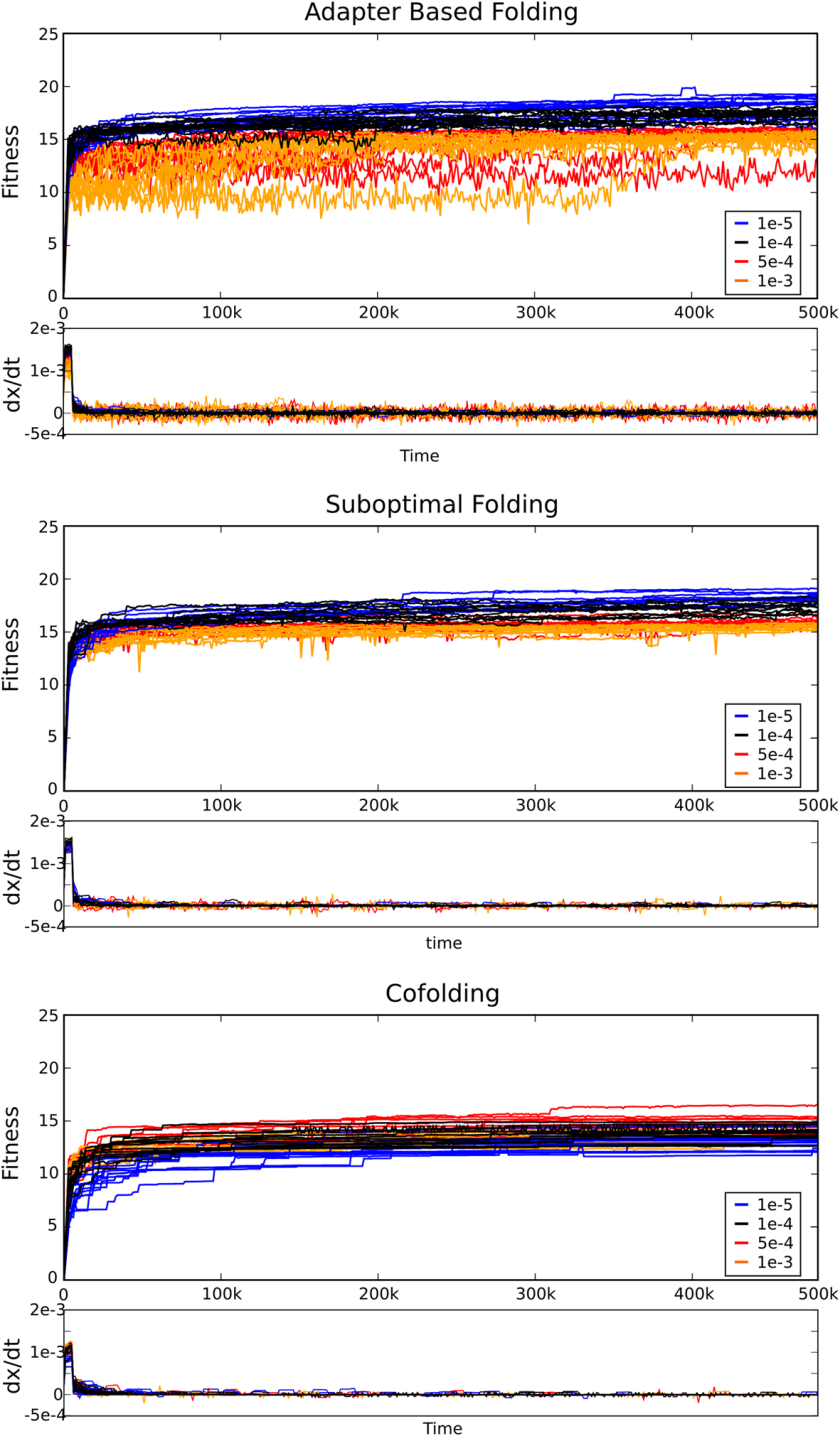
Fitness and derivative of fitness of ten simulations for the different genotype-phenotype mappings. For most simulations, the largest change in fitness takes place within the first 50,000 time-steps. After that, protocells are still evolving, but can be considered to be in evolutionary stable state

For each of the three different genotype-phenotype mappings, we performed ten simulations with different starting populations and random seeds, for each of four mutation rates *µ* = 1×10^−5^, 1 × 10^−4^, 5 × 10^−4^, 1 × 10^−3^. In Fig. 3, all time-series are plotted. Evolution of protocells with suboptimal or adapter-based folding reaches an evolutionary stable state within 20,000 time-steps. For the cofolding regime, the adaptive process is considerably longer, but all studied protocells reach a state of equilibrium within the studied time frame. Note that the adapter-based system keeps a higher rate of change in its evolutionary stable state, compared to suboptimal and cofolding.

**Fig. 4:**
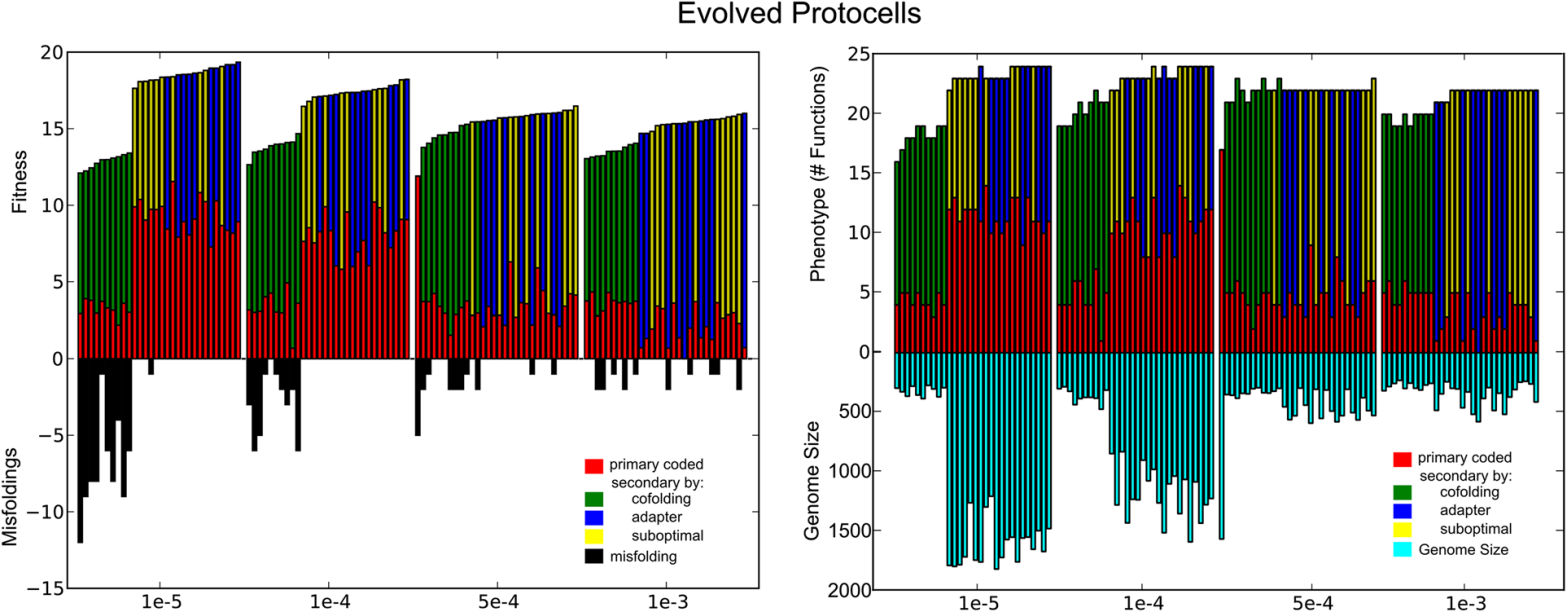
For the mutation rates *µ* = 1×10^−5^, 1 × 10^−4^, 5 × 10^−4^, 1 × 10^−3^, ten simulations for each genotype-phenotype mapping are ranked according to their acquired fitness. Primary coded functions are depicted as *red*; secondary coded structures using cofolding, suboptimal folding, or adapter-based folding are in *green*, *yellow*, and *blue*, respectively. *Left* shows fitness(positive axis) and misfoldings (*black*, negative axis) and *right* shows number of functions (positive axis) and genome size (*cyan*, negative axis). The cofolding regime has the highest numbers of misfoldings and all simulations are ranked in the lower end of the fitness spectrum. However, while fitness and genome size of cofolding seem independent of mutation rates, the number of misfoldings decreases under higher mutation rates with the number of sequences. Note that fitness and misfoldings are explicitly separated over reproduction and lethality, respectively. The simulation under *µ* = 5 × 10^−4^ with the lowest acquired fitness, corresponds to a protocell in the adapter system that does not evolve adapters (see also Fig. 3) (Color figure online)

In Fig. 4, all these simulations are ranked according to the acquired fitness of their LCAs. Note that all simulations under the cofolding map have a considerably lower fitness and more misfoldings than the other two maps. This is most striking under the lowest mutation rates, where the other two mappings are able to exploit the freedom of larger genome sizes, whereas the cofolding mapping only allows limited genome sizes. Fitness of the other two mappings is comparable, and on average ≈18 % higher than cofolding, while under the lowest mutation rate, *µ* = 1×10^−5^, acquired fitness is even ≈31 % higher. Moreover, where suboptimal and adapter-based foldings in most cases are able to successfully avoid misfoldings, cofolding has an average of more than 3 misfoldings per evolved protocell. As a consequence of these high rates of misfoldings in combination with low fitness, populations with cofolding tend to be ≈25 % smaller (data not shown). Interestingly, while fitness under the suboptimal and adapter-based regime is somewhat higher under low mutation rates, fitness under the cofolding regime is independent of mutation rates. Moreover, misfoldings decrease under higher mutation rates. That is, when mutational pressure limits genome size, misfoldings can be avoided.

### Gene Content is Restricted by Mutation Rates

The difference in genotypic variation between the different regimes is considerably larger. The genome sizes in Fig. 4 show that cofolding protocells only maintain small-sized genomes. These small-sized genomes consist on average of less than seven sequences. This is in contrast with adapter-based and suboptimal folding protocells which show a large range in genome size and number of sequences. In the case of adapters, the total number of sequences does not decrease as dramatically as the total genome length. This is because under higher mutation rates more sequences are used to code for adapters. A distinction is made between the part of the genome coding for ‘functions’ and the part used to code for the modification machinery. Hence, when adapters are used, they tend to be small, yet present in considerable amounts (see also de Boer and Hogeweg (2012)).

If we look at the variation over different mutation rates, the differences in cofolding are small over the different mutation rates. Genome size only decreases marginally with mutation rates, as it is already small for low mutation rates. This is due to the selection pressure against misfoldings: the number of foldings increases quadratically with the number of sequences. Indeed, the small decrease in genome size (from eight to six sequences) results in a difference of 13 structures.

Moreover, the ratio of “primary” coded functions to secondary coded functions (see Fig. 4), is observed to be comparable over the different mutation rates, while in the cases of suboptimal and adapter-based folding, a transition can be observed between ‘low’ and ‘high’ mutation rates: under increasing mutational pressure, evolving protocells primarily adapt their coding structure by decreasing the number of sequences.

### Adapters Increase Fitness

**Fig. 5:**
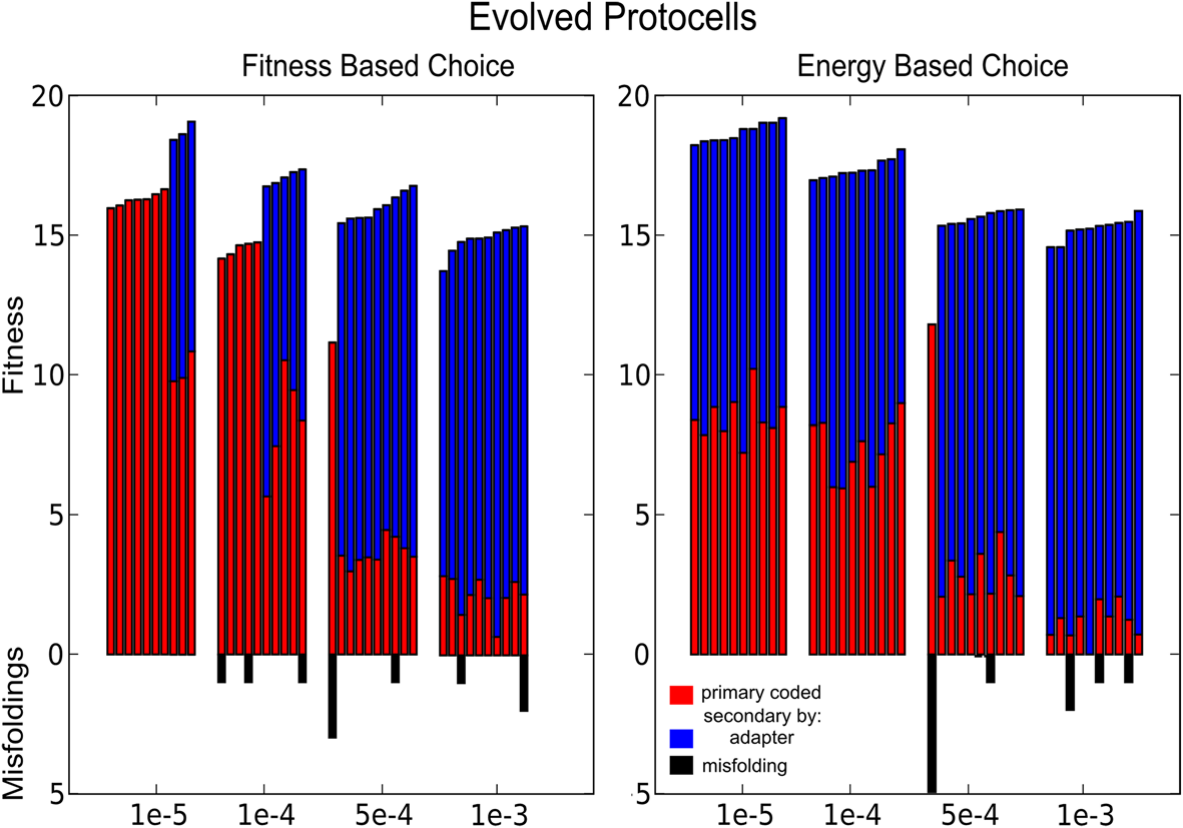
The acquired fitness of twenty simulations with the adapter-based genotype-phenotype mapping is ranked, for the mutation rates *µ* = 1×10^−5^, 1 × 10^−4^, 5 × 10^−4^, 1 × 10^−3^. In one set of simulations, the choice between functional structures is based on energy (*right panel*), in the other set, this choice is based on fitness (*left panel*). Primary coded functions are depicted as *red*; secondary coded structures as *blue*. On the negative axis, the number of corresponding misfolded structures (*black*) is shown. For a given mutation rate, fitness is considerably higher when adapters are evolved (Color figure online)

When adapters are used under low mutation rates (i.e., where they are not needed due to mutational pressure), this allows protocells to gain higher fitness. As a default, when multiple sequences code for the same function, only the structures with the the lowest MFE are considered. Therefore, taking into account the energy given by the binding between adapters and sequences stimulates the use of adapters, i.e., when multiple sequences code for the same function, there is a small bias toward choosing strong adapter-bound sequences. This results in the counter-intuitive observation of Fig. 5 that replicators can actually achieve higher fitness under low mutation rates if the choice between functional structures is based on energy instead of fitness. With such an energy-based choice, adapters evolve in more cases (see also de Boer and Hogeweg (2012)). Note that the actual evolutionary selection criterion and overall scheme are exactly the same. In all cases, if adapters are evolved, fitness is considerably higher.

### Adopting Multiple Coding

In all folding methodologies, high functionality is achieved by the use of multiple coding: at most half of the properly folded structures are primary coded, i.e., are minimal energy structures of single RNA sequences (red vs other colored bars in Fig. 4). At high mutation rates, the number of primary coded functions decreases even further and the system ‘switches’ toward more ‘complex’ coding, within the possibilities given by the different regimes. In the cofolding regime, multiple coding is hard to avoid and, at low mutation rates, leads to many misfoldings. The other regimes can exploit multiple coding while largely avoiding misfoldings.

The increase of multiple coding through adapter-based folding under higher mutation rates is achieved by increasing the use of adapters, as shown in Figs. 4 and 5. This leads to an increase of possible structures produced from a sequence. However, protocells with adapter-based folding have relatively larger genomes under high mutation rates, as almost half of the genome codes for the (partly redundant) adapters.

In the case of suboptimal folding, a single sequence will produce a larger set of functions, as compensation for the genome size being restricted by high mutation rates. While the number of sequences decreases under higher mutation rates, the number of produced structures is comparable. That is, under both *µ* = 1×10^−5^ and *µ* = 1×10^−4^, the average ensemble for all evolved sequences consists of 3.0 and 2.8 different foldings, respectively (as is the case for random sequences), and under the mutation rates *µ* = 5×10^−4^ and *µ* = 1×10^−3^ this increases to a median of 6.0 and 6.6 different suboptimal foldings per sequence.

The cofolding mapping shows no significant decrease in the number of sequences, nor an increase of multiple coding, whereas both suboptimal and adapter-based folding can increase the number of functions coded per sequence without the cost of structurally increased misfoldings by adopting a more dense multiple coding structure on their genome.

The flexibility of these mappings allows for a range of ‘choices’ about how information (approximated by genome size) can be used to code for a phenotype under the different imposed mutation rates. Flexibility is highest for adapter-based folding, where a large range of coding structures is used, allowing it to specifically adapt to the mutation rates it is exposed to. Also the lack of flexibility in the case of cofolding is clear: compared to suboptimal folding and adapter-based folding, cofolding is least able to adapt its genotype-phenotype mapping. It can expand its phenotype, but not prevent the interactions.

### Coding Regimes Combined

**Fig. 6:**
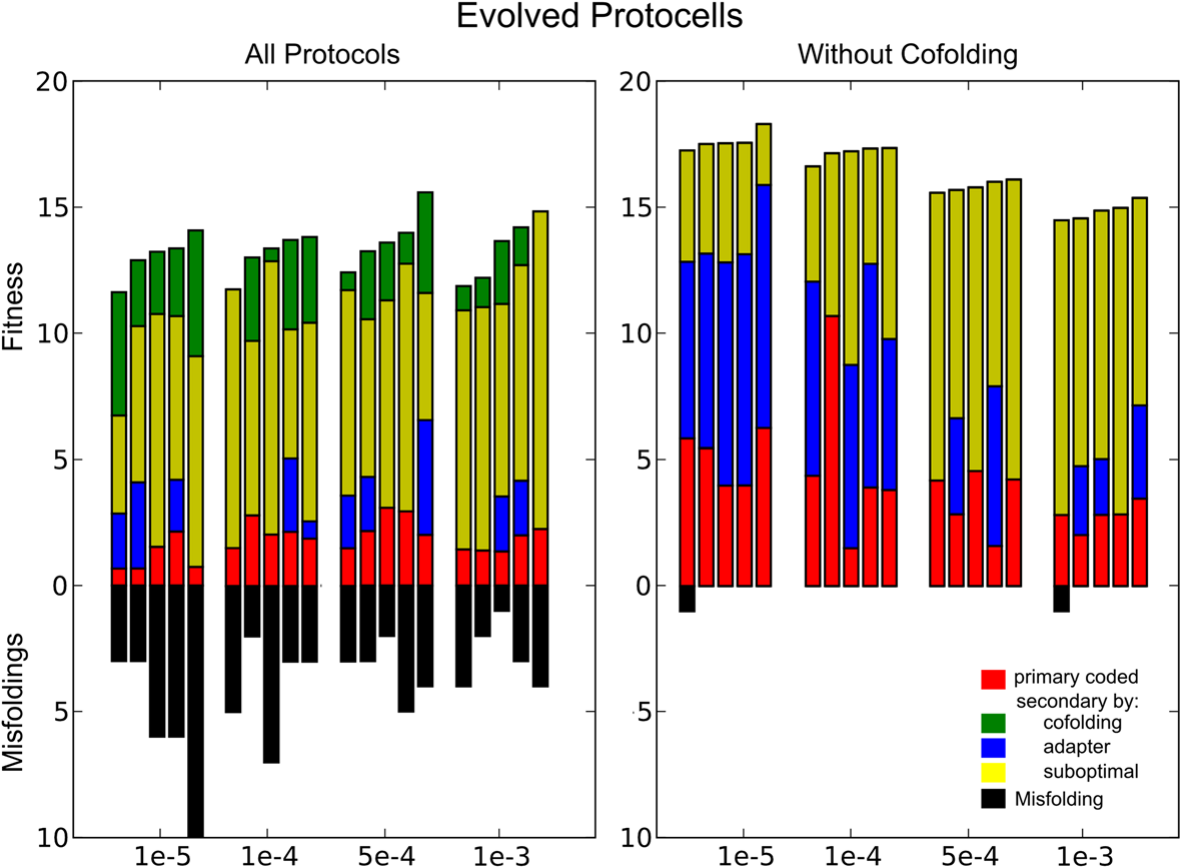
The acquired fitness of five simulations with the possibility of all three mappings(1) and five simulations with adapter based, and suboptimal folding(2) are ranked for the mutation rates *µ* = 1×10^−5^, 1 × 10^−4^, 5× 10^−4^, 1 × 10^−3^. Primary coded functions are depicted as *red*; secondary coded structures using cofolding, suboptimal folding, or adapter-based folding are in *green*, *yellow*, and *blue*, respectively. On the *bottom,* the number of corresponding misfolded structures (*black*) is shown (Color figure online)

An obvious follow-up experiment is to combine the three mappings within a single system. In Fig. 6, we ranked simulations with the possibility of all three mappings side by side with simulations where protocells can use both suboptimal and adapter-based foldings (that is, cofolding is not taken into account). The effect of adding cofolding to the protocol is clear: Overall fitness is lower and the number of misfolded structures increases. That is, because of the selection pressure against misfoldings, genome sizes with cofolding are very small under all mutation rates (data not shown).

Interestingly, adapter-based functionality and fitness are the highest under low mutation rates, while under high mutation rates most functional structures are constructed through suboptimal folding. This is because every sequence has a suboptimal ensemble by default, while adapters have to evolve first. Present from the start, mutational pressure causes the suboptimal ensemble and its multiplicity to be shaped by evolution, rather than the invention of a complex adapter-based system. When genome size is restricted by the cofolding regime as described above, a similar effect can be observed.

### Folding Energies of Functional Structures

**Fig. 7:**
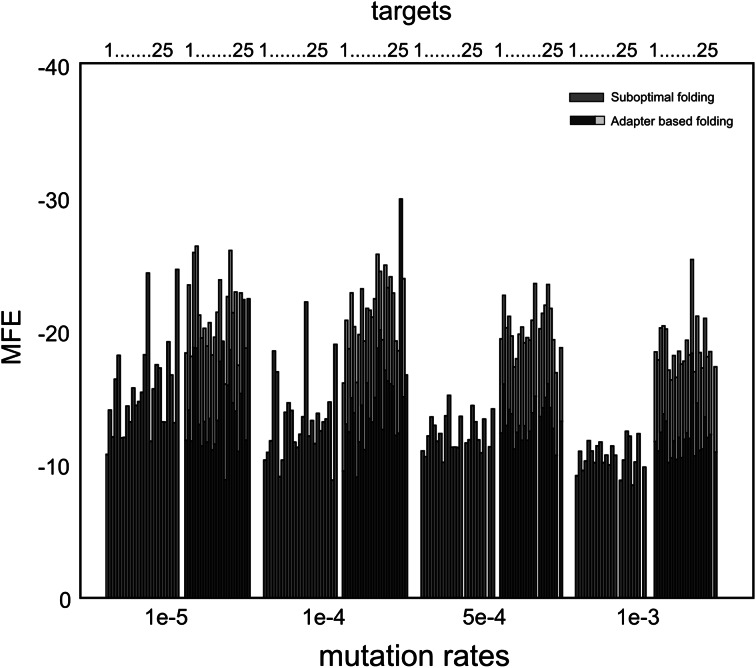
For each mutation rate, the average Minimal Folding Energy of all evolved structures within the target set is shown. In the adapter-based simulations, the average MFE of the binding between adapters is depicted in *yellow*. Note that this is the sequence-adapter interaction only; energies of the base-pairing in the stem of the adapter are not considered. Also note that some targets are more difficult, and therefore, have a smaller sample-size or are not present under certain mutation rates. Even without the adapters, average acquired MFE of adapter-based protocells is stronger. The distributions of energies (without adapter-energies, over all mutation rates) under the two folding regimes differed significantly (*p* = 0.01, Mann–Whitney *U* test). While the most significant difference (*p* = 0.005) is under the highest mutation rate *µ* = 1 × 10^−3^, under *µ* = 1 × 10^−5^ energies did not differ significantly (*p* = 0.44) (Color figure online)

In Fig. 7, we focus on (the separate evolution of) suboptimal and adapter-based folding by comparing the acquired folding energies of the structures. The stability of the evolved functional structures is an important observable to characterize the evolved genotype-phenotype mappings. The stability (energy) of the structures is only used as selection between otherwise equal foldings. Figure 7 shows that this stability in the adapter-based system leads to more stable foldings (i.e., lower energies), whether or not we include the free energy of the adapter-sequence binding (yellow bars). Comparing the free energies obtained without including the adapter-sequence binding, overall the difference is significant (*p* = 0.01, Mann–Whitney *U* test). The most significant difference (*p* = 0.005, Mann–Whitney *U* test) is under the highest mutation rate *µ* = 1 × 10^−3^, while under *µ* = 1 × 10^−5^, energies did not significantly differ (*p* = 0.44, Mann–Whitney *U* test). Thus, although both systems obtain similar fitness, the use of adapters brings lower free energies in the system, providing robust interactions between adapters and sequences, next to the highest flexibility in coding structure.

## Discussion

We studied three different multi-molecule, one-to-many, genotype-phenotype mappings: a map which superimposes combinations of two sequences to cofold, a map where the alternative states of all sequences are considered, and a map which allows for a primitive form of RNA-modification to evolve. The secondary structure is presently the best compromise between theoretical tractability and empirical accessibility (Higgs 2000; Fontana 2002), and it is mostly considered to be a good approximation of the function of a molecule. In our model, fitness depends on the secondary structure, yet to be able to classify molecules into functional and misfolded, the structure of molecules is truncated at the level of the coarse-grained structure (as proposed by Shapiro (1988)). In our opinion, the used classification of ‘functionality’ is adequate, and we think that a more realistic implementation will not lead to qualitative differences.

In conclusion, the intrinsic properties of RNA can cope surprisingly well with the dual constraint of functionality and the penalty on misfoldings in a variety of ways under different mutation rates. As mentioned above, there is no negative effect on the size and variety of the suboptimal ensemble (compare with Ancel and Fontana (2000)). Our simulations show that the phenotypic variation of suboptimal and adapter-based folding is comparable. However, the genotypic variation and flexibility of coding by evolving explicit adapters give more plasticity to cope with the different mutational circumstances. In de Boer and Hogeweg (2012), the advantage of adapters under high mutation rates was emphasized. Now, in addition, we observe that especially under low mutation rates adapters enable protocells to acquire higher fitness and have higher energy foldings overall. Whereas suboptimal folding can only be oppressed, the adapter-based mapping enables protocells to actively ‘choose’ between large genomes and/or multiple coding under different mutation rates. Large genomes are known to be advantageous for evolvability (Knibbe et al. 2007; Cuypers and Hogeweg 2012; de Boer and Hogeweg 2012), and the higher flexibility without loss of functional specificity (see also de Boer and Hogeweg (2012)), has been shown to facilitate evolutionary innovation (Matias Rodrigues and Wagner 2009; Espinosa-Soto et al. 2011).

While misfoldings and fitness are explicitly separated in the model, the cofolding mapping indicates a strong (indirect) correlation between misfoldings and lack of fitness. RNAs with functions dependent on cofolding are expected to evolve much more slowly than RNAs with a function depending only on their own structure (Attolini and Stadler 2005). Indeed, we see exactly this. It is, however, interesting to see that mutation rates have only a slight effect on the cofolding regime. In contrast to the other cases, the cofolding regime performs even better under high mutation rates, with regard to both misfoldings and fitness. With cofolding, flexibility of the genotype-phenotype mapping is low and the number of sequences (and genome size) is inflexible. As a result, protocells with cofolding have a high number of misfoldings, low evolvability, and lower fitness than the other two mappings.

An ‘ideal’ simulation involves the concepts of the three mappings combined. Which strategy will dominate? This cannot be entirely answered with our current model, because functionality and molecular interactions within protocells are collapsed over their lifespan and resources are neglected. However, our results show, in the form of cofolding, that too many (forced) interactions restrict evolvability.

In the combined case, multiple coding is most often accomplished by suboptimal folding. However energy-wise, adapters do have an advantage over suboptimal folding. That is, the observed minimal energies from structures acquired with adapters are not affected by increasing mutation rates. This suggests a possible role for ‘simple’ RNA-adapters and the evolutionary exploitation of such binding-induced function-alterations. This suggestion is reinforced by the parallels which can be observed between our RNA-adapters and the widespread use of riboswitches, that are able to regulate several processes by changing their conformational states (Vitreschak et al. 2004; Serganov and Patel 2007; Montange and Batey 2008; Zhang et al. 2010).

On the other side of the spectrum, a very interesting question rises with the observed limitation of the cofolding mapping: how are the unwanted interactions between molecules avoided in a (proto)cell? In this light, comparable to our results, it has been shown for proteins that interactions pose a general structural (and energy binding) constraint, through specific interacting interfaces, which have to be maintained, while other interactions have to be avoided (Deeds et al. 2007).

We conclude that evolved multiple coding can increase fitness and evolvability both at low and at high mutation rates. This potential is best realized in the adapter-based regime that allows many functional foldings with high stability and an absence of misfoldings, with small genomes at high mutation rates and large genomes at low mutation rates.

However, if multiple coding is hard to avoid or shape, as is the case in the cofolding regime, genome size is restricted also at low mutation rates. This leads to a relatively low number of functional foldings and many misfoldings. Interestingly, when genome size is restricted due to high mutation rates functionality is retained and misfoldings can be avoided.
